# Redundant nerve roots in lumbar spinal stenosis: inter- and intra-rater reliability of an MRI-based classification

**DOI:** 10.1007/s00234-019-02337-3

**Published:** 2019-12-14

**Authors:** Luca Papavero, Carlos J. Marques, Jens Lohmann, Thies Fitting, Kathrin Schawjinski, Nawar Ali, Hauke Hillebrand, Rainer Maas

**Affiliations:** 1 Clinic for Spine Surgery, Schoen Clinic Hamburg Eilbek, Academic Hospital of the University of Hamburg, Dehnhaide 120, 22081 Hamburg, Germany; 2 Science Office of the Orthopedic and Joint Replacement Department, Schoen Clinic Hamburg Eilbek, Dehnhaide 120, 22081 Hamburg, Germany; 3 Non-Medical PhD Program, Faculty of Medicine, University of Hamburg, Hamburg, Germany; 4 Department of Radiology at the Schoen Clinic Hamburg Eilbek, Academic Hospital of the University of Hamburg, Dehnhaide 120, 22081 Hamburg, Germany; 5 Radiology Office Raboisen 38, Hamburg, Germany

**Keywords:** Redundant nerve roots, Lumbar spinal stenosis, Neurogenic claudication, Magnetic resonance imaging, Classification

## Abstract

**Purpose:**

Patients with central lumbar spinal stenosis (LSS) have a longer symptom history, more severe stenosis, and worse postoperative outcomes, when redundant nerve roots (RNRs) are evident in the preoperative MRI. The objective was to test the inter- and intra-rater reliability of an MRI-based classification for RNR.

**Methods:**

This is a retrospective reliability study. A neuroradiologist, an orthopedic surgeon, a neurosurgeon, and three orthopedic surgeons in-training classified RNR on 126 preoperative MRIs of patients with LSS admitted for microsurgical decompression. On sagittal and axial T2-weighted images, the following four categories were classified: allocation (A) of the key stenotic level, shape (S), extension (E), and direction (D) of the RNR. A second read with cases ordered differently was performed 4 weeks later. Fleiss and Cohen’s kappa procedures were used to determine reliability.

**Results:**

The allocation, shape, extension, and direction (ASED) classification showed moderate to almost perfect inter-rater reliability, with kappa values (95% CI) of 0.86 (0.83, 0.90), 0.62 (0.57, 0.66), 0.56 (0.51, 0.60), and 0.66 (0.63, 0.70) for allocation, shape, extension, and direction, respectively. Intra-rater reliability was almost perfect, with kappa values of 0.90 (0.88, 0.92), 0.86 (0.84, 0.88), and 0.84 (0.81, 0.87) for shape, extension, and direction, respectively. Intra-rater kappa values were similar for junior and senior raters. Kappa values for inter-rater reliability were similar between the first and second reads (*p* = 0.06) among junior raters and improved among senior raters (*p* = 0.008).

**Conclusions:**

The MRI-based classification of RNR showed moderate-to-almost perfect inter-rater and almost perfect intra-rater reliability.

## Introduction

**Fig. 1:**
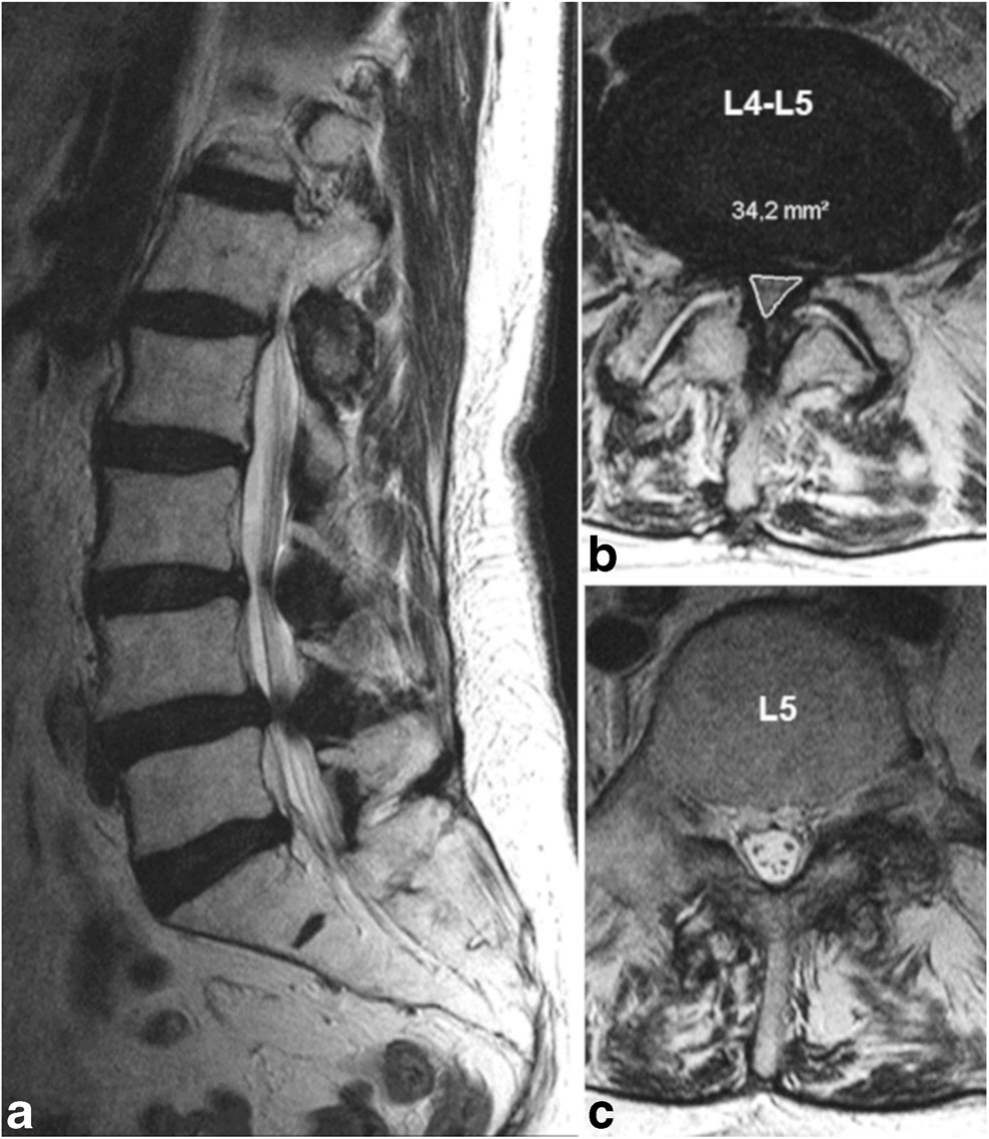
**a** Sagittal T2-weighted images (WI) with an almost normal course of the cauda nerve roots (CNRs) despite a stenotic level grade D at L4/L5 (**b**) according to Schizas et al. [17] in the axial slice. **c** The CNRs are distributed throughout the cross-sectional area of the dural sac (positive nerve roots sedimentation sign). No evidence of redundant nerve roots (RNR-)

**Fig. 2:**
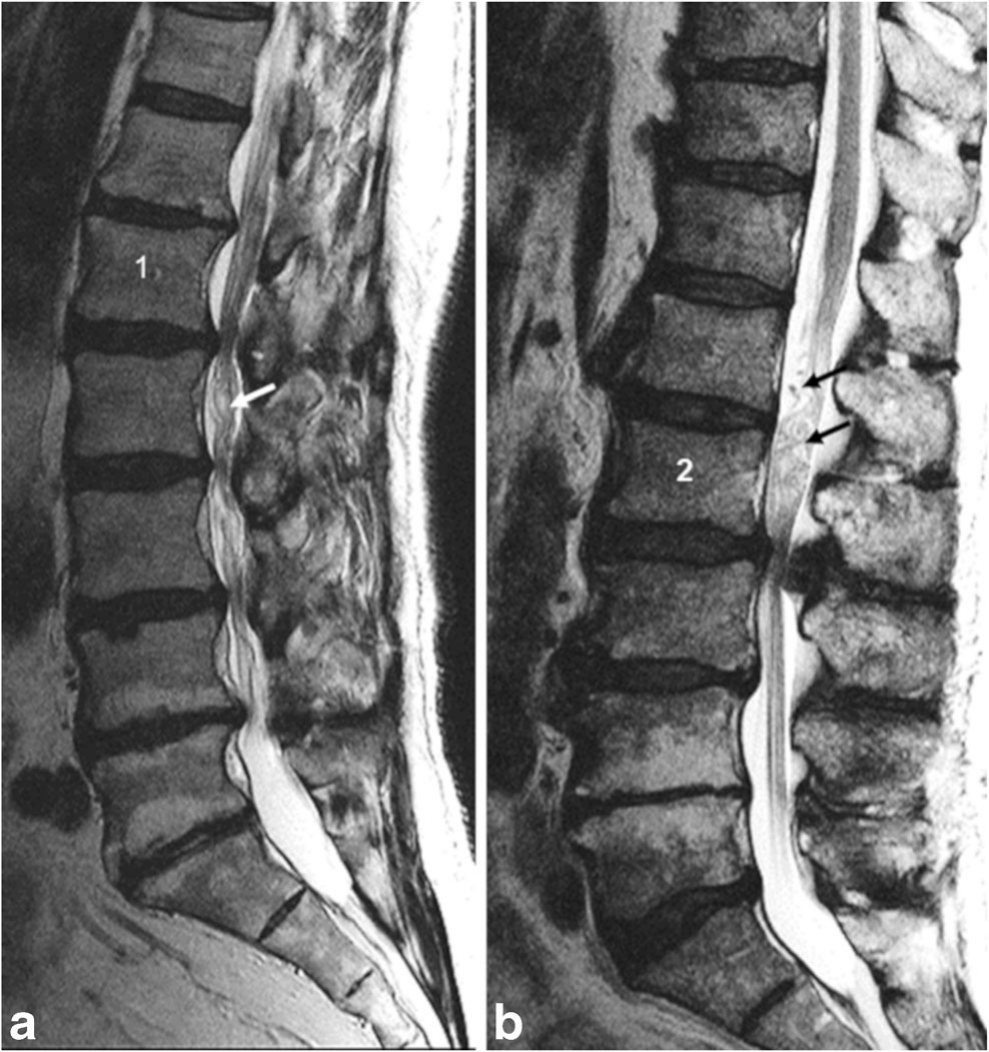
**a** Sagittal T2WI with stretched cauda nerve roots cranially and serpentine RNRs (white arrow) caudally from the key stenotic level (KSL) at L1/L2. **b** KSL at L2/L3 with stretched CNRs caudally and loop-shaped RNRs cranially (black arrows)

Decompression treatment for lumbar spinal stenosis (LSS) is the most common spine surgery in patients older than 65 years in the USA [1]. In roughly 60% of patients with LSS scheduled for surgery, the natural course of cauda nerve roots (CNR) remains unaltered, even in the presence of severe stenosis (Fig. 1). In the remaining 40% of patients, redundant nerve roots (RNRs) of the cauda equina are evident on preoperative magnetic resonance images (MRIs) [2–5]. RNRs were first described by Verbiest [6] in 1954, and they were named 14 years later by Cressman and Pawl [7]. RNRs were described as thickened, buckling, coiled CNRs with a serpentine (Fig. 2a) or loop-like (Fig. 2b) shape in sagittal T2-weighted images (T2WI) [8].

Little is known about the clinical significance of RNRs in patients with LSS. A recent meta-analysis revealed that, among patients with LSS, those that showed evidence of RNRs were older, had a longer symptom history, and presented higher degrees of lumbar stenosis preoperatively than those without RNRs. Moreover, after decompression surgery, patients with RNRs showed worse clinical scores and lower recovery rates than those without RNRs [9]. A study on potential RNR predictors demonstrated that patients with LSS with evidence of RNRs on the preoperative MRI were older had a shorter lumbar spine canal, displayed stenosis at more levels, and had more severe stenosis, compared to patients without RNRs [10].

Yokoyama et al. studied patients with LSS and found that most RNRs resolved postoperatively, but some did not. Among patients with unresolved postoperative RNRs, functional outcome remained poor, even when the dural sac was sufficiently expanded. Furthermore, among patients with LSS, those with loop-shaped RNRs performed worse than those with serpentine-shaped RNRs [11].

Although the etiology and pathogenesis of RNRs are only partially understood, they appear to indicate more advanced LSS stages, and they are a negative prognostic factor [9].

In daily radiological practice, MRI reports of patients with LSS mostly describe changes in bony structures, discs, facet joints, and yellow ligament. A validated classification system for RNRs could facilitate descriptions of changes in the CNR and could provide clinicians with additional relevant information. To the best of our knowledge, a validated classification system for RNRs does not exist [12]. The aim of the present study was to test the inter-rater and intra-rater reliability of an MRI-based classification system for RNRs in LSS.

## Materials and methods

### Study design

For this retrospective study, we acquired database data to investigate inter- and intra-rater reliability. This study was developed in accordance with the “Guidelines for Reporting Reliability and Agreement Studies” (GRRAS) [13]. The reporting follows the STROBE Statement guidelines [14].

The Ethics Commission of the Federal State of Hamburg, Germany, approved the research proposal (File PV 5767). Informed consent was not necessary, because the data were collected and treated anonymously.

### Sample

A sample size calculation was based on the work by Rotondi and Donner [15]. First, we assumed the proportions of the three items in the category “direction” were 0.10, 0.20, and 0.70. We determined that 126 MRIs were required to ensure that a two-sided 95% confidence interval (CI) for a target kappa value (*k*) of 0.80 did not exceed the lower bound of 0.70.

We identified data for 126 patients with LSS (47 females) that underwent decompression surgery. The mean age was 74.2 ± 9 years. Women (mean age 76.4 ± 8.9 years) were 3.4 years older (*P* = 0.03) than men (mean age 72.9 ± 8.9 years). All patients had evidence of RNRs and underwent decompression surgery in the same institution, between December 2012 and August 2016.

Inclusion criteria were as follows: symptomatic central LSS that required surgical decompression without fixation; available preoperative MRIs of at least 1.5 Tesla (T), including sagittal and axial T2-WIs, stored in the picture archive and communication system of the institution; and evidence of RNRs. Exclusion criteria were as follows: a previous history of lumbar spine surgery; scoliosis or a vertebral slip that required fixation; and congenital, traumatic, infectious, or neoplastic diseases of the lumbar spine.

### The raters

Three senior raters (one neuroradiologist, one orthopedic surgeon, and one neurosurgeon with 15, 10, and 35 years of experience, respectively) and three junior raters (orthopedic surgeons in training) independently classified all RNRs on the MRIs.

### The MRI-based definition of RNRs

**Fig. 3:**
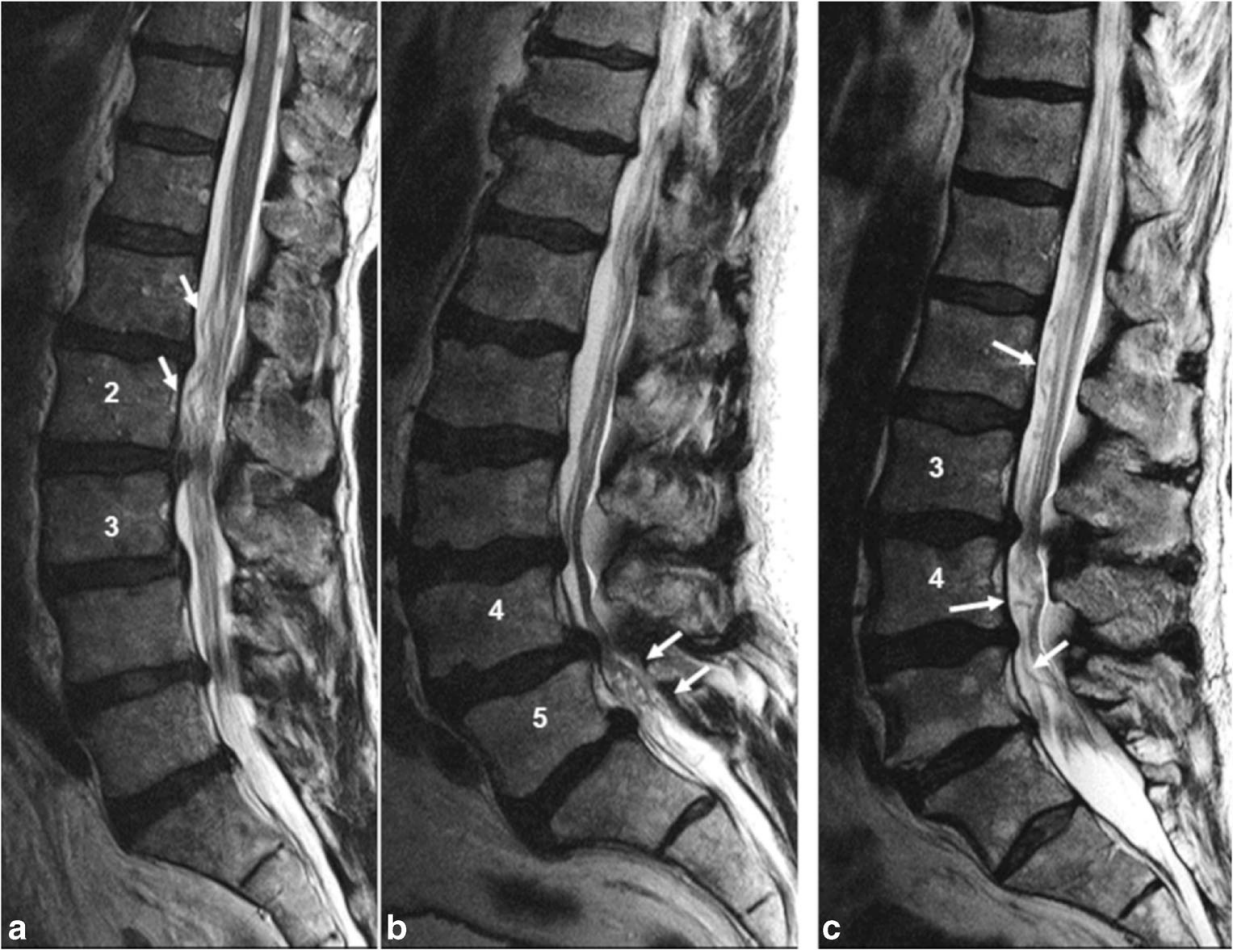
**a** Sagittal T2WI with RNRs cranial, **b** caudal, and **c** cranial-caudal from the KSL. The ASED notation would be as follows: **a** RNR+: L2/L3.S.1+.cr; **b** RNR+: L4/L5.L.1.ca; and **c** RNR+: L3/L4.L.1+.cc

An MRI was defined as presenting RNRs when the key stenotic level (KSL) altered the natural course of the CNR. In most cases, CNRs were straight on one side of the KSL and serpentine or loop-shaped on the opposite side. Rarely, the CNR looked either serpentine or coiled on both sides of the KSL. Most RNRs were located cranial to the KSL, some were located caudal to the KSL, and a few were located cranial-caudal to the KSL [16] (Fig. 3).

### The ASED classification of RNRs

The morphologies of RNRs were classified into four categories, including allocation, shape, extension, and direction (ASED). The ASED classification system is described in Table 1. Examples are illustrated in Fig. 3.

**Table 1:** ASED classification for RNR

ASED	Category definition	Items	Item definition	Notation
Allocation	Refers to the key stenotic level (KSL). The KSL shows the switch between straightened cauda nerve roots (CNR) and RNR or, shows adjacent cranio-caudal RNR. When a doubt between two potential KSL occurs, the most stenotic level (with the smallest CSA) is defined as KSL.	L1/L2		Notation of the KSL (i.e., L3/L4)
		L2/L3		
		L3/L4		
		L4/L5		
		L5/S1		
Shape	Refers to the shape of RNR. This category comprises two items.	Serpentines	Serpentines are present when a sinusoidal deflection (complete crest-trough wave) of the majority of CNR occurs within the height of a vertebral body without any horizontalization of the involved roots.	S
		Loops	Loops are present when at least in two different areas dots or horizontal roots in the sagittal T2WI-slice were combined with tortuous, coiled roots in the axial T2WI- slice. Mixed serpentine and loop findings are scored as loops.	L
Extension	Refers to the length of RNR. This category comprises two items.	1	When RNR extend up to one vertebral height adjacent to the KSL they are notated with “1”.	1
		1+	When RNR extend beyond one vertebral height they are notated with “1+.” Cranio-caudal RNRs are always notated as 1+.	1+
Direction	Refers to the localization of RNR in relation to the KSL. This category comprises three items.	Cranial	RNR are only present cranially from the KSL	cr
		Caudal	RNR are only present caudally from the KSL	ca
		Cranial-caudal	RNR are present cranially and caudally from the KSL	cc

*RNR* redundant nerve roots, *KSL* key stenotic level, *CNR* cauda nerve roots, *CSA* cross sectional area

### Procedures

The classifications of shape, extension, and direction depend on the allocation. Therefore, all raters first classified the allocations independently. Then, we calculated the inter-rater kappa value for allocation. In 22 cases, discordances occurred between at least two raters. These cases were discussed and resolved by consensus. Thereafter, all raters independently classified the RNRs on the 126 MRIs, according to the definitions for shape, extension, and direction, considering the previously agreed KSL allocations. In the second read, performed 4 weeks later, after altering the order of cases, the allocation values were used from the first read. These were fixed after calculating the inter-rater kappa values. Because the allocation category was only rated once, intra-rater kappa values for this category were not calculated.

In addition to the ASED classification, all raters classified the LSS grade with the qualitative grading system, based on the root-to-cerebrospinal fluid relationship described by Schizas et al. [17].

### Statistical analysis

To determine the proportion of agreement, the Fleiss kappa (*k*) was used to assess inter-rater reliability. The Fleiss kappa is an extension of Cohen’s kappa; it can be used when nominal categories are assessed by more than two raters [18]. In this study, Fleiss kappa values were calculated for junior raters, senior raters, and for all 6 raters, for both reads. Cohen’s kappa (*k*) was used to calculate intra-rater reliability [19]. Mean kappa values for intra-rater reliability were calculated separately for junior and senior raters. The *k* values were categorized to reflect different levels of agreement, as follows: ≤ 0.00: poor, 0.00–0.20: slight, 0.21–0.40: fair, 0.41–0.60: moderate, 0.61–0.80: substantial, and ≥ 0.81: almost perfect [20].

To determine whether inter-rater kappa values differed significantly between the two reads, we performed paired sample *t* tests. Inter-rater kappa values for the 1st and 2nd reads were compared between junior and senior raters with independent sample *t* tests. The assumptions associated with the different tests were verified previously.

We performed all statistical analyses with IBM SPSS software version 21 for Macintosh (IBM Corp. Armonk, New York). The 0.05 level of probability was set as the criterion for statistical significance.

## Results

The results for inter-rater reliability are presented in Table 2. The ASED classification showed moderate-to-almost perfect inter-rater reliability. In the 1st read, all 6 raters achieved kappa values (95% CI) that ranged from 0.56 (0.51, 0.60), for extension, to 0.86 (0.83, 0.90), for allocation. The kappa values of junior raters did not change significantly between the 1st and the 2nd reads (*P* = 0.06). In contrast, senior raters achieved higher inter-rater kappa values in the 2nd read than in the first read (*P* = 0.008). When all raters (*n* = 6) were considered, there was no significant difference between inter-rater kappa values of both reads (*P* = 0.5).

**Table 2:** Inter-rater reliability for the ASED classification of RNR

ASED categories/items	1st read			2nd read		
	Junior raters (*n* = 3)	Senior raters (*n* = 3)	All raters (*n* = 6)	Junior raters (*n* = 3)	Senior raters (*n* = 3)	All raters (*n* = 6)
	Kappa (95% CI)	Kappa (95% CI)	Kappa (95% CI)	Kappa (95% CI)	Kappa (95% CI)	Kappa (95% CI)
Allocation	0.89 (0.82, 0.96)	0.82 (0.70, 0.94)	0.86 (0.83, 0.90)	0.89 (0.82, 0.96)	0.82 (0.70, 0.94)	0.86 (0.83, 0.90)
Shape	0.66 (0.56, 0.76)	0.62 (0.52, 0.72)	0.62 (0.57, 0.66)	0.59 (0.49, 0.69)	0.63 (0.53, 0.73)	0.59 (0.55, 0.64)
Extension	0.57 (0.47, 0.67)	0.60 (0.49, 0.70)	0.56 (0.51, 0.60)	0.53 (0.43, 0.63)	0.68 (0.58, 0.78)	0.59 (0.55, 0.64)
Direction (overall)	0.64 (0.57, 0.72)	0.74 (0.64, 0.82)	0.66 (0.63, 0.70)	0.62 (0.55, 0.70)	0.82 (0.74, 0.90)	0.65 (0.62, 0.69)
Cranial (cr)	0.74 (0.64, 0.84)	0.80 (0.70, 0.90)	0.76 (0.72, 0.81)	0.68 (0.58, 0.78)	0.89 (0.79, 1)	0.75 (0.71, 0.80)
Caudal (ca)	0.67 (0.56, 0.77)	0.80 (0.70, 0.91)	0.72 (0.67, 0.76)	0.63 (0.53, 0.73)	0.83 (0.73, 0.93)	0.68 (0.63, 0.72)
Cranio-caudal (cc)	0.48 (0.37, 0.58)	0.38 (0.28, 0.48)	0.39 (0.35, 0.44)	0.53 (0.43, 0.63)	0.54 (0.44, 0.64)	0.42 (0.38, 0.47)
LSS grade^a^	0.77 (0.67, 0.87)	0.64 (0.54, 0.74)	0.69 (0.65, 0.74)	0.68 (0.58, 0.78)	0.76 (0.67, 0.86)	0.67 (0.62, 0.71)

Values are Fleiss kappa with 95% confidence intervals for junior raters, senior raters, and for all six raters, for the 1st and 2nd reads

^**a**^Grade of LSS according to Schizas et al. [17]

The results for intra-rater reliability are presented in Table 3. The ASED classification showed almost perfect intra-rater reliability. For junior raters, the mean kappa values ranged from 0.83 (0.76, 0.90), for shape, to 0.86 (0.82, 0.90) for extension. Senior raters achieved similar mean kappa values, except in the shape category (*k* = 0.90 [0.88, 0.92]).

**Table 3:** Intra-rater reliability for the ASED classification of RNR

	Junior raters				Senior raters			
	Rater A	Rater B	Rater C	Mean Kappa	Rater D	Rater E	Rater F	Mean Kappa
	Kappa (95% C.I.)	Kappa (95% C.I.)	Kappa (95% C.I.)	Kappa (95% C.I.)	Kappa (95% C.I.)	Kappa (95% C.I.)	Kappa (95% C.I.)	Kappa (95% C.I.)
Shape	0.91 (0.84, 0.98)	0.90 (0.83, 0.91)	0.68 (0.55, 0.81)	*0.83(0.76, 0.90)*	0.96 (0.93, 0.99)	0.89 (0.82, 0.97)	0.85 (0.78, 0.92)	0.90 (0.88, 0.92)
Extension	0.92 (0.85, 0.99)	0.90 (0.83, 0.91)	0.76 (0.61, 0.91)	0.86 (0.82, 0.90)	0.87 (0.80, 0.94)	0.81 (0.72, 0.90)	0.90 (0.83, 0.97)	0.86 (0.84, 0.88)
Direction	0.91 (0.85, 0.97)	0.90 (0.85, 0.95)	0.76 (0.67, 0.85)	0.85 (0.85, 0.89)	0.91 (0.86, 0.96)	0.84 (0.77, 0.91)	0.78 (0.69, 0.87)	0.84 (0.81, 0.87)
LSS grade^a^	0.97 (0.93, 1)	0.91 (0.84, 0.98)	0.78 (0.67, 0.89)	0.88 (0.83, 0.93)	0.95 (0.90, 1)	0.54 (0.39, 0.69)	0.85 (0.76, 0.94)	0.78 (0.67, 0.89)

Values are Cohen’s kappa with 95% confidence interval for the single raters and mean Cohen’s Kappa with 95% confidence interval for the three junior and three senior raters

^**a**^Grade of LSS according to Schizas et al. [17]

Inter-rater reliability for LSS grade was substantial in the first read for all raters (*k* = 0.69 [0.65, 0.74]). The intra-rater reliabilities were substantial (*k* = 0.78 [0.67, 0.89]) and almost perfect (*k* = 0.88 [0.83, 0.93]), for senior and junior raters, respectively.

In 95.6% of cases, the KSL was located in the central part of the lumbar spine, with *n* = 56 (44.4%) at L3/L4, *n* = 35 (27.8%) at L4/L5, and *n* = 31(24.6%) at L2/L3. In four cases (3.2%), the KSL was located at L1/L2, but it was never located at L5/S1.

The severity of LSS was scored according to the classification purposed by Schizas et al. [17]. We observed “surgical” grade C in 94 (75%) cases and grade D in 30 (24%) cases. We observed stenosis grade B in two cases (1%).

## Discussion

Previous reports have shown that patients with LSS that displayed RNRs in preoperative MRIs had worse postoperative outcomes compared to patients without evidence of RNRs. Those findings suggested that RNRs comprise a negative prognostic factor [9, 11, 21].

To the best of our knowledge, a validated MRI-based RNR classification has not been established. Here, we presented an ASED classification for RNRs. Six raters with different grades of experience independently scored 126 MRIs with ASED classifications. They exhibited moderate to almost perfect inter-rater and almost perfect intra-rater reliabilities. These results indicated that the ASED classification could be used in daily radiological practice to complete MRI reports on patients with LSS.

We classified the LSS severity grade with the well-known grading system of Schizas et al. [17]. They assessed its intra- and inter-rater reliabilities with 57 axial T2 MRIs of patients with LSS. Those authors reported average kappa values of 0.44 ± 0.17 and 0.65 ± 0.14 for inter- and intra-rater reliabilities, respectively. Raters from the originating study unit achieved higher kappa values (*k* = 0.67 ± 0.08 and *k* = 0.77 ± 0.06, respectively). Our study results confirmed their results with a sample that was twofold larger; we found *k* = 0.69 and *k* = 0.78 for inter- and intra-rater reliabilities, respectively (Tables 2 and 3). Moreover, like Schizas et al., we also found that inter-rater kappa values were lower than intra-rater kappa values. However, in their study, neither inter-rater nor intra-rater reliability achieved mean kappa values above substantial agreement.

The ASED classification comprised four categories with 12 items for scoring RNRs on T2 sagittal and axial MRIs. This classification is more complex than the LSS grading system [17]. Our results indicated that the ASED was reliable and could be used in clinical practice.

In the 1st read, the inter-rater kappa values of all 6 raters for the direction category were 0.76 (0.72, 0.81) for cranial, 0.72 (0.67, 0.76) for caudal, and 0.39 (0.35, 0.44) for cranio-caudal. However, kappa values are affected by the distribution of data across the categories (prevalence bias). The frequency distribution that we observed across the three items in the direction category was *n* = 84 (66.7%) for cranial, *n* = 35 (27.8%) for caudal, and *n* = 7 (5.6%) for cranio-caudal. This unequal distribution influenced the kappa values of the respective items, as outlined previously by Byrt et al. [22].

Our results confirmed the surgical relevance of the KSL, the key element of the ASED classification. When rating the KSL, the raters were blinded to the surgical levels. Interestingly, all except four KSLs (97%) were decompressed. Moreover, in 42 patients (33%), a second level was decompressed, and in eight other patients (6%), two additional levels were decompressed. Of the four cases that showed a discrepancy between the KSL and the operated level, two patients displayed more stenosis at the operated level than at the KSL, and the two other patients displayed disc herniations associated with a stenotic level adjacent to the KSL.

In our daily practice, we nicknamed the KSL “the switch level,” because, in 119 out of 126 patients, at the KSL, the CNR “switched” from a straight course to a RNR shape. In the other seven patients, the KSL was embedded in a cranio-caudal RNR.

In addition, we confirmed the clinical relevance of the LSS grade classification of Schizas et al. [17]. We observed “surgical” grade C in 94 (75%) patients and grade D in 30 (24%) patients.

Physicians that care for patients with LSS expect the MRI report to answer the following questions: Is there any LSS, and how severe is it? Which level(s) and anatomic structures are involved? In our opinion, to counsel patients with LSS about adequate treatment, clinicians also need information about the degree of compromise at the CNR. Thus, the MRI report should answer the question: Are RNRs present, and what is their shape, extension, and direction? A previous study by Min et al. [23] examined associations between the relative length of RNRs and the symptom duration and recovery rates; they found moderately positive (*r* = 0.38) and strongly positive (*r* = 0.53) correlations, respectively. Ono et al. [4] reported that a group with higher numbers of loop RNRs had a higher mean duration of neurological symptoms and worse preoperative walking ability, compared to a group with higher numbers of serpentine RNRs. To investigate these issues further, a validated RNR classification system is necessary.

In a previous review, Nogueira-Barbosa et al. [24] suggested that radiologists should examine MRIs for RNRs in the cauda equina and, when applicable, describe those findings in the MRI report. We share that opinion, and to facilitate the descriptions, we have presented the ASED classification.

Although imaging should not influence the surgical indication [12, 25], our results pointed out the relevance of imaging in surgical planning. We defined the MRI quality of the sample by choosing a field strength of at least 1.5 T. However, in daily practice, different observers have different perceptions of the image quality of 1.5 T MRIs.

For a long time, researchers have debated the validity of the signal-to-noise ratio as an objective quality measure for biomedical images [26]. In the present study, different image resolutions may have led to differences in scoring. This was a study limitation.

In conclusion, we demonstrated that the ASED classification for RNRs was reliable and feasible. It should be included in the MRI report for patients with LSS that display evidence of RNRs.
